# Resuscitation on the pitch with cardiac massage and on-site AED

**DOI:** 10.1007/s12471-018-1109-1

**Published:** 2018-04-04

**Authors:** N. M. Panhuyzen-Goedkoop, J. J. Piek

**Affiliations:** 1 AMC Heart Center, Academic Medical Center, Amsterdam, The Netherlands; 2 Sports Medical Center Papendal Arnhem, Arnhem, The Netherlands; 3 Radboud University Medical Center, Nijmegen, The Netherlands

We thank our colleague Calle for his comments on our editorial ‘Resuscitation on the pitch’ [1, 2]. Calle describes correctly the problem of resuscitation of an athlete who collapsed during exercise. He also mentions that the ‘European Council of Resuscitation (ERC) still endorses the need for rescue breaths in all cardiac arrest cases’ [1]. We do agree on the airway-breathing-circulation sequence of resuscitation according to the ERC guidelines in the overall population, such as spectators and personnel in the stadium. However, we challenged the ERC guidelines when an athlete collapses suddenly during exercise. The cause of this sudden collapse is almost always a cardiac cause, i. e. sudden cardiac arrest (SCA). Therefore, we would like to recommend to start immediately with cardiac massage and defibrillation on-site using an AED [2, 3]. While the bystanders continue cardiac massage and defibrillation, another bystander can check on the airway and breathing of the victim. If the airway and breathing are incorrectly interpreted that the person is still alive, needless minutes are lost without restoring the circulation [3]. Therefore, bystanders should immediately start and continue without interruption cardiac massage and defibrillation.

Another point Calle discusses is an obvious mistake of the number of cases of ventricular tachycardia and fibrillation (VT/VF) in the overall population being higher than the annual incidence of sudden cardiac death (SCD). Perhaps we did not quote the two referred papers clearly in our editorial. However, the VT/VF includes the survivors of SCD, meaning survivors of SCA due to a shockable rhythm of VT/VF.
